# Association between maximum occlusal force and 3-year all-cause mortality in community-dwelling elderly people

**DOI:** 10.1186/s12903-016-0283-z

**Published:** 2016-09-01

**Authors:** Toshimitsu Iinuma, Yasumichi Arai, Michiyo Takayama, Yukiko Abe, Tomoka Ito, Yugaku Kondo, Nobuyoshi Hirose, Nobuhito Gionhaku

**Affiliations:** 1 Department of Complete Denture Prosthodontics, Nihon University School of Dentistry, Tokyo, Japan; 2 Center for Supercentenarian Medical Research, Keio University School of Medicine, Tokyo, Japan; 3 Center for Preventive Medicine, Keio University School of Medicine, Tokyo, Japan

**Keywords:** Very elderly people, Bite force, All-cause mortality, Healthy life expectancy

## Abstract

**Background:**

Among the very elderly, poor oral health reduces life expectancy. In this study, differences in the magnitude of the maximum occlusal force (MOF) in the very elderly were examined in terms of effects on all-cause mortality in a 3-year follow-up.

**Methods:**

We evaluated 489 community-living elderly individuals aged 85 years or older. MOF was measured using an occlusal force measuring device, and participants were classified into three groups according to gender- and dental status-sensitive tertiles. Demographic variables, cognitive, physical function, psychological status, oral health, comorbidity, and blood chemistry factors were assessed. One-way analyses of variance, *χ*^2^ tests, and the Kruskal-Wallis test were used for statistical analyses. The relationship between MOF tertiles and 3-year all-cause mortality was examined using a multivariate Cox model analysis after adjusting for confounding factors.

**Results:**

MOF tertiles were significantly associated with cognitive impairment, number of teeth, limitations on chewable foods, handgrip strength, timed up-and-go test, and diabetes mellitus. During the follow-up period, 74 subjects died. Subjects with the highest MOF had a significantly lower mortality rate than other groups (log rank *P*  =  0.031). In the univariate Cox model, MOF tertiles were independently associated with a lower risk of death (HR = 0.69, 95 % CI = 0.51–0.91). Even after adjusting for various confounders in the multivariate Cox model (Model 1), MOF was independently associated with a lower risk of death (HR = 0.67, 95 % CI = 0.50–0.91). In model 2, we added handgrip strength as a confounder and found that the HR for MOF was attenuated (HR = 0.73, 95 % CI = 0.54–0.99), but still statistically significant.

**Conclusions:**

In a cohort of the very elderly, MOF was independently associated with all-cause mortality after adjusting for various health issues. Moreover, this independent association remained after a further adjustment for handgrip strength; however, the HR was attenuated. This suggests that MOF and handgrip strength may share a common mechanism of a general decrease in muscle strength, possibly sarcopenia, which is a significant cause of mortality in the very old.

## Background

Many studies of oral health status have examined factors such as number of teeth, oral hygiene, denture use status, saliva secretion, and masticatory function to clarify any relationship with mortality in the elderly [1–7]. In addition, masticatory function has been studied in terms of nutritional analysis from the diet, functional analysis by muscle activity involved in chewing, and analysis of the maximum occlusal force (MOF) [8–10].

There are several ways to measure masticatory function and chewing ability with objective indicators. These indicators include evaluation methods using questionnaires, a chewing efficiency determination method using samples (peanuts, chewing gum, and gummy jelly), and measuring MOF values [11–13]. Among them, MOF is a safe and easy way for subjects to be measured. Thus, it has been used in many studies. In addition, measurement of MOF is straightforward.

Though a significant relationship between MOF and physical function has been observed [10], few studies have investigated the relationship between MOF and mortality. Iwasaki et al. [14] investigated survival over 13 years in 70-year-old elderly people who participated in the Niigata study, where they divided subjects, first separated by gender, into tertiles according to maximum bite force to investigate its relationship with mortality. Male participants in the lower maximum bite force group showed increased risk of all-cause mortality compared with those in the upper maximum bite force group. The range of MOF is gender-sensitive and affected by the presence or absence of teeth [15–17]. Furthermore, as the average life span increases, further studies on very elderly people aged 85 years or older are required. Despite the increase in comorbidities and worsening of physical status, psychological status, and nutrition status [18–20] with increasing age, we hypothesized that MOF in the very elderly would be associated with their mortality independent of general health status.

In this study, we investigated the influence of the magnitude of MOF, classified by gender and number of teeth, based on survival at 3 years. Furthermore, we examined the relationships between MOF and physical and oral function. If our hypothesis is correct, MOF can be considered an indicator of the relationship between oral health and mortality in very elderly people.

## Methods

### Study population

The study analyzed data from participants in The Tokyo Oldest Old Survey on Total Health (TOOTH) study, which is an epidemiological survey involving very elderly people living in the Tokyo metropolitan area.

We recruited 542 subjects [236 men, 306 women; mean age ± SD, 87.8 + 2.2 (range 85–102) years] for a medical and dental survey. The participants were randomly selected from inhabitants between March 2008 and November 2009 [21].

Fifty-three participants without MOF measurements were excluded from the analyses for the following reasons: pain on biting, non-use of dentures, and current dental treatment. Consequently, data of 489 subjects were analyzed. The study protocol was reviewed and the survey was approved by the ethics committees at Nihon University School of Dentistry (No. 2003–20, 2008) and Keio University School of Medicine (No. 19–47, 2007). The TOOTH study has been registered in the UMIN-Clinical Trial Registry as UMIN-CTR ID UMIN000001842.

### MOF

The MOF was measured using an occlusal force-measuring device (Occlusal Force-Meter GM10; Nagano Keiki, Tokyo, Japan, with a measuring range of 0 ~ 1000 N and accuracy of ±1 N) using a standard procedure. We calculated MOF as the average of three measurements of the first molar on the functional side. The subjects were seated in an unsupported natural position, and the users’ dentures were inserted at that time. The intra-class correlation coefficients (ICC) for the reproducibility of the MOF measurements for intra- and inter-class reliability were 0.98 and 0.97, respectively [10].

### MOF tertile classification

We classified the participants according to MOF tertile using the following three steps because MOF is sensitive to gender and dental status. First, we classified participants into four categories depending on gender and the presence or absence of teeth (edentulous male, dentulous male, edentulous female, and dentulous female). Then each category was classified into three groups based on MOF value (highest, middle, and lowest groups). Finally, to form three groups distinguished only by MOF, we merged the above four categories. (e.g., the MOF lowest tertile group included participants with MOFs in the lowest tertile within each group: edentulous male, dentulous male, edentulous female, and dentulous female). Accordingly, MOF could minimize the influence of gender, and presence or absence of teeth.

### Oral health assessment

In face-to-face interviews and dental examinations, trained dentists assessed subjects’ oral health status in terms of the number of remaining teeth, number of chewable foods, and degree of oral hygiene.

For the food intake questionnaire, 15 kinds of food were selected to evaluate chewing ability, and were categorized into three groups according to hardness and texture. Limitation of chewing ability was defined as being unable to eat one or more foods. In this study, we counted the total number of remaining teeth (range 0–32). Tongue surface plaque was examined to evaluate the degree of oral cleaning [22].

### Demographics and general health assessment

At the same time as the dental examination, trained geriatricians assessed the subjects’ demographic variables and general health, including medical conditions and physical functional status. Education was dichotomized according to whether participants had graduated from high school. Smoking and drinking were dichotomized in terms of whether participants had never or had ever smoked and whether they had ever or had never consumed alcohol, respectively. Ten indices of basic activities of daily living (ADL) were assessed using the Barthel index. A disability in ADL was defined as having a disability on one or more indices. Cognitive function was evaluated using the Mini-Mental State Examination (MMSE). Body mass index (BMI) was measured as an anthropometric parameter. The World Health Organization-5 (WHO-5) well-being index was used to assess psychological status [23]. To obtain the medical history, medical examinations conducted by geriatricians, personal interviews regarding available documentation including discharge summary and medication list, and personal interviews with the caregiver/proxy were used.

Self-reported medical conditions were classified according to the International Classification of Diseases, 10th Revision. Using non-fasting blood samples, we measured the plasma albumin (ALB) and C-reactive protein (CRP) concentrations using standard assay procedures. We used commercial ELISA kits (Quantikine HS, Human IL-6; R&D Systems, Minneapolis, MN) in duplicate to measure plasma interleukin-6 (IL-6) levels. The inter-assay CV for IL-6 was 9.43 %.

### Physical function and activity

To assess lower extremity performance, the timed up-and-go test, which has been validated in older adults [24], was used. The test was performed by two examiners to prevent falls or injuries. A handheld dynamometer (Tanita 6103; Tanita, Tokyo, Japan) was used to measure handgrip strength of the dominant hand, and the average of twice measurements was calculated.

The physical activity was evaluated by asking whether participants walked more than 60 min per day.

### Outcomes

The outcome of interest was death from any cause. All of the participants were followed by telephone contact or mail survey every 12 months to identify mortality. We also investigated the cause-specific mortality, including cancers, cardiovascular disease, pneumonia, and other causes. The survivors after 36 months were surveyed using the same protocol as at baseline.

### Statistical analysis

Data are expressed as means [standard deviations (SDs)] or medians [interquartile ranges (IQRs)] for continuous variables or percentages for categorical variables, or were log-transformed for statistical analyses. The Kruskal–Wallis test was used to evaluate differences in continuous variables at baseline, and the chi-square test was used to assess categorical variables. Longitudinal analyses were performed using Kaplan–Meier survival curves according to MOF tertiles. The level of significance for prognostic results was defined as log-rank *P* < 0.05. Then, univariate and multivariate Cox proportional hazards regression models were used to identify the relative risk of mortality. First, biological and behavioral factors known to be associated with mortality (MOF, age, smoking and drinking status, cognitive impairment [MMSE < 24], ADL disability, low body mass index [<18.5], physical function [handgrip strength], physical activity [total walking time], oral hygiene [tongue plaque], nutrition [limited ability to chew foods], psychological status [WHO-5], and comorbidities [ischemic stroke, coronary heart disease, cancer], ALB, CRP, and IL-6) were assessed using the hazard ratio (HR) in univariate models. Those with significant associations (*P* < 0.05) were entered into the multivariate model. To eliminate multicollinearity between CRP and IL-6, these values were entered into the model separately. In all analyses, the level of statistical significance was set at *P* < 0.05. Analyses were performed using SPSS ver. 22.0 (SPSS, Chicago, IL, USA).

## Results

**Fig. 1:**
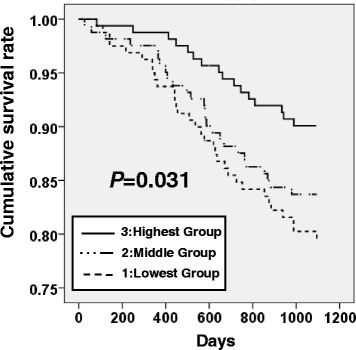
MOF tertiles and 3-year all-cause mortality. Kaplan-Meier survival curves for the three maximum occlusal force groups (*P* = 0.031). Cumulative survival rate increased progressively in association with an increase in maximum occlusal force

The MOF tertiles are shown in Table 1. The median MOF was 14.0 kgf (IQR: 7.9–23.8 kgf) in males (219 participants) and 9.9 kgf (IQR: 5.4–16.7 kgf) in females (270 participants). There was a significant difference between the genders (*P* < 0.001). Regarding the presence or absence of teeth, the median MOF was 13.8 kgf (IQR: 8.0–22.3 kgf) in the dentulous (349 participants) and 7.7 kgf (IQR: 4.0–12.5 kgf) in the edentulous (140 participants); the difference was significant (*P* < 0.001). Baseline characteristics of subjects by MOF tertiles are shown in Table 2. MOF tertiles were significantly associated with cognitive impairment, number of teeth, limited ability to chew foods, handgrip strength, timed up-and-go test, and prevalence of diabetes mellitus (*P* = 0.001, *P* < 0.001, *P* < 0.001, *P* = 0.006, *P* = 0.014, and *P* = 0.047, respectively). During the 3-year follow-up period, 74 participants died (22 of cancer, 22 of cardiovascular disease, 16 of pneumonia, 11 of other causes, and three of unknown causes); four declined the follow-up survey. We plotted Kaplan–Meier survival curves for the cumulative incidence of all-cause mortality according to oral function (Fig. 1). The survival curve of MOF tertiles showed that subjects with the highest MOF had a significantly lower risk of all-cause mortality (log rank *P* = 0.031). Table 3 presents HRs and 95 % CI from Cox proportional hazards models for all-cause mortality. In the univariate Cox model, MOF tertiles (lowest as a reference) were associated with a lower HR of death (HR = 0.69, 95 % CI = 0.51–0.91). In addition, age, ADL disability, handgrip strength, total walking time, WHO-5 score, ALB, IL-6, and CRP were significantly associated with all-cause mortality. When we adjusted for age, ADL disability, total walking time, WHO-5, ALB, IL-6, and CRP in the multivariate Cox model (model 1), MOF was independently associated with a ~0.7-fold lower risk of 3-year survival (HR = 0.67, 95 % CI = 0.50–0.91). In model 2, we added handgrip strength as a confounder and found that the HR for MOF was attenuated (HR = 0.73, 95 % CI = 0.54–0.99), although the estimate was still statistically significant.

**Table 1:** Tertiles of MOF across age and dentulous status

	Maximum occlusal force groups (tertile)			
	All	1(lowest)	2	3(highest)
Characteristics	*n*=489	*n*=163	*n*=163	*n*=163
Maximum occlusal force, median [IQR], kgf				
Male dentulous (*n*=167)	15.7[8.9–26.1]	7.3[5.5–9.0]	15.7[13.1–18.8]	31.4[26.0–41.4]
Male edentulous (*n*=52)	11.2[4.7–16.3]	4.0[3.1–4.7]	11.2[8.4–12.4]	18.8[16.3–24.7]
Female dentulous (*n*=182)	11.9[7.2–18.6]	5.1[3.3–7.3]	11.9[10.1–14.2]	22.3[18.6–28.8]
Female edentulous (*n*=88)	6.6[3.6–10.9]	2.8[1.9–3.7]	6.6[5.4–8.1]	12.3[10.9–18.0]

**Table 2:** Baseline characteristics of participants according to tertiles of MOF

	MOF groups (tertile)				
	All	1(lowest)	2	3(highest)	*p* ^*^
Characteristics	*n*=489	*n*=163	*n*=163	*n*=163	
Demographics					
Age, mean (SD)	87.3(2.2)	87.3(2.4)	87.5(2.3)	87.1(2.0)	0.264
Higher education, %^c^	26.5	26.1	27.2	26.1	0.968
Smoking, %^c^	40.6	35.8	44.3	41.6	0.294
Drinking, %^d^	48.8	45.0	49.4	52.2	0.431
Oral health status					
Number of teeth, median [IQR]	7.0[0.0–18.0]	5.0[0.0–11.0]	6.0[0.0–17.0]	15.0[0.0–24.0]	<0.001^a^
Limitation of chewable foods, %	46.9	60.7	50.0	30.1	<0.001
Tongue plaque, %	41.3	44.8	40.5	38.7	0.514
General health assessment					
MMSE, median [IQR]^e^	27[25–29]	27[23–29]	27[25–29]	27[25–29]	0.072^a^
MMSE<24, %^e^	17.8	26.7	12.4	14.2	0.001
ADL disability, %^f^	22.8	24.4	24.1	20.0	0.581
BMI, mean (SD)	21.5(3.1)	21.1(3.0)	21.6(2.9)	21.9(3.4)	0.119
BMI<18.5, %	17.0	19.0	14.7	17.3	0.583
Psychological status					
WHO-5, median [IQR]^d^	19.0[15.0–22.0]	19.0[15.0–22.0]	19.0[14.0–22.5]	20.0[17.0–22.0]	0.195^a^
Medical history, %					
Ischemic stroke	11.9	11.7	12.9	11.0	0.872
Coronary heart disease	11.2	11.0	10.4	12.3	0.866
Hypertension^g^	59.3	58.6	57.1	62.1	0.526
Diabetes mellitus	18.4	15.3	24.5	15.3	0.047
Cancer	18.6	19.0	19.0	17.8	0.947
Biochemical					
Albumin, g/dL (SD)^g^	4.1(0.3)	4.1(0.3)	4.1(0.3)	4.1(0.2)	0.372
IL-6, pg/ml, median [IQR]^g^	1.69[1.29–2.46]	1.75[1.32–2.47]	1.72[1.33–2.54]	1.63[1.21–2.43]	0.568^b^
CRP, mg/dL, median [IQR]^g^	0.09[0.04–0.18]	0.08[0.04–0.19]	0.10[0.04–0.21]	0.08[0.04–0.15]	0.594^b^
Physical functions					
Handgrip strength, median[IQR], kgf	19.3[15.5–24.0]	18.3[15.0–22.8]	19.0[15.5–23.3]	20.5[16.0–25.5]	0.006^a^
Time up & go test, median[IQR], second^h^	13.1[10.9–16.2]	13.8[11.3–18.7]	13.2[10.9–15.4]	12.7[11.0–15.2]	0.014^a^
Physical activity					
Total walking time>60 min/day, %^d^	37.2	36.3	40.0	35.2	0.649

^*^ *P* values were calculated for categorical covariates using chi-square test, whereas P variables were calculated using one way analysis of variance (ANOVA) for continuous variables unless otherwise indicated. ^a^Calculated using nonparametric Kruskal-Wallis test. ^b^Calculated using ANOVA after logarithmic transformation

^c-h^Data available for ^c^472, ^d^477, ^e^484, ^f^482, ^g^486, and ^h^432 people, respectively

**Table 3:** Hazard risk from univariate and multivariate cox models for mortality

	Univariate model			Multivariate model 1			Multivariate model 2		
Characteristics	HR	95 % CI	*p*-value	HR	95 % CI	*p-*value	HR	95 % CI	*p*-value
MOF (tertile)	0.69	[0.51–0.91]	0.010	0.67	[0.50–0.91]	0.010	0.73	[0.54–0.99]	0.040
Age	1.14	[1.06–1.23]	<0.001	1.11	[1.03–1.20]	0.006	1.08	[1.01–1.17]	0.038
Smoking	0.83	[0.51–1.35]	0.454						
Drinking	0.88	[0.55–1.41]	0.594						
Cognitive impairment (MMSE<24)	1.32	[0.76–2.30]	0.331						
ADL disability	1.70	[1.04–2.77]	0.035	1.11	[0.64–1.92]	0.714	1.05	[0.61–1.81]	0.866
BMI <18.5	1.15	[0.64–2.05]	0.645						
Handgrip strength (tertile)	0.72	[0.54–0.96]	0.026				0.58	[0.41–0.82]	0.002
Total walking time (>60min/day)	0.51	[0.30–0.88]	0.015	0.60	[0.34–1.06]	0.079	0.66	[0.37–1.16]	0.148
Limitation of chewable foods	1.46	[0.93–2.32]	0.103						
Tongue plaque	0.92	[0.58–1.46]	0.725						
WHO-5 (tertile)	0.72	[0.53–0.97]	0.032	0.77	[0.56–1.06]	0.114	0.79	[0.58–1.09]	0.152
Ischemic stroke	1.32	[0.70–2.51]	0.390						
Coronary heart disease	1.40	[0.74–2.66]	0.303						
Cancer	1.53	[0.91–2.58]	0.108						
ALB (tertile)	0.64	[0.47–0.87]	0.005	0.77	[0.55–1.08]	0.124	0.80	[0.57–1.11]	0.183
IL-6 (tertile)^a^	1.33	[1.00–1.76]	0.048	1.22	[0.90–1.66]	0.191	1.23	[0.90–1.66]	0.190
CRP (tertile)^a^	1.45	[1.09–1.94]	0.011	1.30	[0.96–1.76]	0.096	1.32	[0.97–1.80]	0.077

Model 1: Adjusted for age, ADL disability, MOF, Total walking time, WHO-5, ALB, IL-6, and CRP

Model 2: Adjusted for age, ADL disability, MOF, Total walking time, WHO-5, ALB, IL-6, CRP, and handgrip strength

^a^IL-6 and CRP were entered separately in the multivariate model. Tertile: lowest was a reference

## Discussion

This prospective study investigated community-dwelling very elderly people age ≥85 years, after minimizing the influence of effects of gender and the number of teeth present and adjusted for potential confounders. The results indicated that the risk of death in the subpopulation with the highest MOF conferred a 0.7-fold lower risk of death in three years versus the lowest MOF group (tertile). This suggests that the association between MOF and mortality is independent of gender, dental status, physical activity, psychological status, comorbidities, and serum levels of albumin and inflammatory biomarkers. Moreover, this independent association remained after a further adjustment for handgrip strength; however, the hazard ratio of MOF was attenuated. These results suggest that MOF and handgrip strength may at least partly share a common mechanism in the general decrease in muscle strength, possibly sarcopenia, a significant contributor to mortality in the very old. Moreover, we expect our results will be useful for the systematic evaluation of life expectancy in the very elderly.

Many studies of the elderly have investigated relationships between mortality and nutrition, lifestyle, comorbidities, physical status, and physical function [25–28]. Furthermore, factors concerning oral status, inflammation, and function have been investigated for the same purpose [29–31]. Recently, one study investigated the association between MOF and mortality in older Japanese adults, and reported that MOF was independently associated with all-cause mortality in males [14]. However, studies on the relationship between MOF and mortality are very limited, because it is generally considered that gender, age, remaining number of teeth, use of denture, and face morphology affect one’s bite force [32, 33]. In fact, in the present study, significant differences were observed between men and women and between dentulous and edentulous subjects. This finding is partly consistent with the results of observational studies, which have demonstrated gender-sensitive effects on bite force and masticatory muscle thickness and their interactions [34, 35]. In this study, we took these factors into account by separating subjects into four categories. The participants were divided into three groups by MOF, gender, and dentulous versus edentulous. Even considering these factors, the 3-year all-cause mortality for the highest MOF group was significantly lower than the others. One reason why MOF affects mortality may be that a reduction of muscle activity from sarcopenia due to aging causes a decline in the skeletal muscle mass in the whole body [36]. In addition, a decrease in skeletal muscle function in the very elderly causes falls and leads to a reduction in ADL [37, 38]. Ling et al. [20] reported on 555 people who were followed for a period of 9.5 years in the Leiden 85-plus study. In their results, 80 % of the participants died, and handgrip strength was a predictor of all-cause mortality in the oldest old population. Raadsheer et al. [32] concluded that the size of the jaw muscles was significantly related to the size of the limb muscles; that is, a decrease in muscle mass of the whole body, due to sarcopenia, affected the muscle activity of the jaw muscles that express the bite force. In our study, MOF showed a significant association with handgrip strength and the timed up-and-go test, which are both physical functional measurement items. In addition, from the results obtained by adding handgrip strength as a confounding factor in the Cox hazard analysis, the correlation between MOF and death was affected. Based on these results, the pathogenesis of sarcopenia caused by aging could have the same impact on bite force, extremity muscle strength, and handgrip strength, and these might represent a significant mortality force among the very old. Hence, MOF is an important indicator of sarcopenia in the oral area in the very elderly. Thus, dental health professionals, there is a need for careful observation of very elderly concerning changes in the value of MOF in everyday clinical practice, because decreases in MOF may be associated with worsening physical health. Furthermore, the effects of aging on muscle activity may also be due to an attenuation of muscle strength (dynapenia) [39]. Further research is necessary to determine any such association.

MOF might affect meals and the nutritional value associated with food intake in the very elderly. In the present study, as an index of the variety of food intake in the diet of the very elderly, the proportion of those with no limit on food acceptance was significantly higher in the highest MOF group than in the other groups. However, MOF showed no significant correlation with serum levels of albumin or BMI <18.5, which are indices of malnutrition. From these results, we believe that a decline in MOF may limit the diversity of food acceptance rather than total calories from food. In this regard, there is substantial evidence that a decline in oral function is associated with a suboptimal diet that is low in fruits and vegetables, leading to micronutrient deficiencies [40]. Thus, there is a need to analyze any connection between MOF and nutrient intake.

This study had several limitations. First, the number of participants and areas of research were limited, as our study targeted very elderly people who lived in metropolitan Tokyo. Therefore, a larger-scale, separate cohort study that considers race, eating habits, and economic conditions should be conducted. Furthermore, to explore the underlying biological mechanism, it is important to study the relationship between MOF and cause-specific mortality. However, this study did not include a sufficient number of individuals over a long enough period to analyze cause-specific mortality. Second, we classified the participants by the number of teeth as dentulous and edentulous. However, there is a need to subdivide by number of teeth and details. Third, we analyzed food variety. There is a need to examine the relevance of micronutrients. Finally, it is necessary to make a concrete plan to attempt to improve MOF in the very elderly, and also to advocate for their needs in an aging society.

## Conclusions

In a cohort of the very elderly, MOF was independently associated with all-cause mortality after adjustment for various health elements, including ADL disability, physical activity, psychological status, and levels of albumin, interleukin-6, and C-reactive protein. These results suggest that MOF can be considered an important indicator of the relationship between oral health and mortality in very elderly people. Moreover, further adjustment for handgrip strength affected the HR of MOF, suggesting that age-related sarcopenia could at least partly mediate the association between MOF and mortality. The results of this study encourage dental health professionals to pay attention to MOF in the very elderly because decreases in MOF may reflect worsening physical health.

## Abbreviations

ADL: Activities of daily living
ALB: Albumin
BMI: Body mass index
CI: Confidence interval
CRP: C-reactive protein
HR: Hazard ratio
IL-6: Interleukin-6
IQR: Interquartile range
MMSE: Mini-Mental State Examination
MOF: Maximum occlusal force
SD: Standard deviation
TOOTH: The Tokyo Oldest Old Survey on Total Health
WHO-5: The World Health Organization-5 well-being index
